# Identifying Positive Adaptive Pathways in Low-Income Families in Singapore: Protocol for Sequential, Longitudinal Mixed-Methods Design

**DOI:** 10.2196/11629

**Published:** 2019-02-01

**Authors:** Esther Chor Leng Goh, Wan Har Chong, Jayashree Mohanty, Evelyn Chung Ning Law, Chin-Ying Stephen Hsu, Jan De Mol, Leon Kuczynski

**Affiliations:** 1 Department of Social Work Faculty of Arts and Social Sciences National University of Singapore Singapore Singapore; 2 National Institute of Education Nanyang Technological University Singapore Singapore; 3 School of Social Work University of Windsor Ontario, ON Canada; 4 Department of Paediatrics Yong Loo Lin School of Medicine National University of Singapore Singapore Singapore; 5 Department of Paediatrics Khoo Teck Puat–National University Children's Medical Institute National University Health System Singapore Singapore; 6 Faculty of Dentistry National University of Singapore Singapore Singapore; 7 Faculty of Psychology and Educational Sciences University of Louvain Louvain Belgium; 8 Department of Family Relations and Applied Nutrition University of Guelph Guelph, ON Canada

**Keywords:** low-income families, family agency, adaptive pathways, trajectories

## Abstract

**Background:**

This study aims to examine the adaptive process of children and mothers from multistressed low-income families in Singapore. It aims to bridge the knowledge gap left by existing poverty studies, which are predominately risk focused. Through a sequential longitudinal mixed-methods design, we will differentiate children and mothers who demonstrate varied social, developmental, and mental health trajectories of outcomes. Through utilizing the Latent Growth Curve Model (LGCM), we aim to detect the development and changes of the positive *Family Agency* and adaptive capacities of these families over time. The construct of *Family Agency* is underpinned by the theoretical guidance from the Social Relational Theory, which examines child agency, parent agency, relational agency, and the interactions among these members. It is hypothesized that positive *Family Agency* within low-income families may lead to better outcomes. The key research questions include whether the extent of positive *Family Agency* mediates the relationship among financial stress, resource utilization, home environment, and parental stress.

**Objective:**

The study elucidates the *Family Agency* construct through interviews with mother-child dyads. It also aims to understand how financial stress and resources are differentially related to home environment, parent stress, and parent and child outcomes.

**Methods:**

In phase 1, 60 mother-child dyads from families receiving government financial assistance and with children aged between 7 and 12 years will be recruited. In-depth interviews will be conducted separately with mothers and children. On the basis of 120 interviews, a measurement for the construct of *Family Agency* will be developed and will be pilot tested. In phase 2a, a longitudinal survey will be conducted over 3 time points from 800 mother-child dyads. The 3 waves of survey results will be analyzed by LGCM to identify the trajectories of adaptation pathways of these low-income families. In addition, 10 focus groups with up to 15 participants in each will be conducted to validate the LGCM results.

**Results:**

This project is funded by the Social Science Research Thematic Grant (Singapore). The recruitment of 60 mother-child dyads has been achieved. Data collection will commence once the amendment to the protocol has been approved by the Institutional Review Board. Analysis of phase 1 data will be completed by the end of the first quarter of 2019, and the first set of results is expected to be submitted for publication by the second quarter of 2019. Phase 2 implementation will commence in the second quarter of 2019, and the project end date is in May 2021.

**Conclusions:**

Findings from this study can potentially inform social policy and programs as it refines the understanding of low-income families by distinguishing trajectories of adaptive capacities so that policies and interventions can be targeted in enhancing the adaptive pathways of low-income families with children.

**International Registered Report Identifier (IRRID):**

PRR1-10.2196/11629

## Introduction

### Background

The Singapore government has, in recent years, shifted its policies aiming to rectify the trend of income divide and make Singapore a more inclusive society. This is reflected in the national budget in the past decade [1], introducing an increased number of redistributive measures of wealth to families from the lowest stratum. These include Workfare, which was implemented in 2007, ComCare—a government-funded short-term financial assistance scheme introduced in 2005— as well as progressive tax rebates and utility subsidies [2]. Together with civic charity efforts through nonprofit organizations [3], there are initial signs of narrowing of the rapid income divide as reflected in slightly declined Gini coefficient rates in recent years from a peak in 2007 [4]. Such interventions may avert the potential formation of an underclass as warned by some economists [5]. This shift in the government’s fundamental approach to poverty and welfare should be acknowledged as crucial steps toward social inclusion, preventing a weakening of social cohesion and the consequent wide range of social ills if inequality grows unchecked [6].

Little is known, however, as to whether the new redistributive schemes have strengthened the organic adaptive capacities of the lowest quintile (bottom 20%) of households. In an attempt to bridge this gap, this proposed study seeks to examine adaptive microprocesses and approaches that low-income families used in their daily interactions and transactions to navigate the challenges and new redistributive opportunities in coping with financial constraints. This proposed study aims to advance the construct of family adaptation using an innovative perspective posited by the Social Relational Theory (SRT) [7]. SRT is a dynamic systems framework used in studying family dynamics as it pertains to children’s socialization and development. SRT’s core constructs, which emphasize that family members, both children and parents, are active agents. The construct of *Family Agency* is conceptualized as the agency of family members acting individually on behalf of the family or collaboratively to manage their financial challenges. It is hypothesized that positive *Family Agency* mediates the financial difficulties and outcomes. In other words, families with positive *Family Agency* are better able to manage the financial challenges they face that result in better social, emotional health, and mental health outcomes for children and mothers of these low-income families [8-11].

Some of the dynamic constructs of *Family Agency* can be tested using existing instruments: *children as agent* in poor families by Children’s Family Influence Behavior [12], *relational agency* between family members in dealing with poverty by Influence in Families Questionnaire [13], and Network of Relationships Inventory [14]. The dimensions of *Family Agency* that lack existing instruments as proxy include children’s contributions to their families and the bidirectional influences between mothers and children that enhance Family Agency. These dimensions will be distilled from in-depth observations of low-income families in Singapore in phase 1 of this proposed study. We will then formulate measurement items that will be the best proxy for these constructs.

### Impact of Poverty on Children and Families

Economic deprivation is 1 pervasive environmental factor that cuts deeply to affect families and children in diverse ways. An overview of the literature suggests that families with low socioeconomic status do not only face significant physical and mental risks but also expose their children to various developmental vulnerabilities that have come to predict their developmental trajectories in major domains of functioning: physical and mental health, educational performance and achievement, emotional and behavioral well-being, as well as executive function and social competence [15-18]. These effects are cumulative, pervasive, and can impact one’s life course in the long run and possibly transmitted intergenerationally [15,19].

Chronic economic strain influences children’s future life experiences and outcomes [17,20]. Research has shown that families with fewer economic, educational, and psychological resources are less able to provide a home environment that supports their children’s cognitive growth and development and prepare them for school [21,22]. The income effect is a potential mediating or moderating pathway through the materials and services parents provide for their children [19].

Economically deprived families are often compounded by attendant life stressors such as poor housing, chronic unemployment or unstable employment, single parenthood, teenage pregnancy, family violence, parental incarceration, and family members’ physical or mental health issues [15,19,23,24]. Parenting stress arising from these compounded stressors then mediates the effects of poverty on child adjustment [25,26]. A recent Singapore study [27] concurs with major studies that show poorer mental health conditions by primary caregivers compared with those of the typical population. Between 14% and 31% reported *severe* to *extremely severe* scores measured with the Depression, Anxiety and Stress Scale (DASS) [28], above the reported national statistics of 10% for depressive and anxiety disorders in Singapore.

A body of research has built up to reveal the long reach of poverty on the psychological well-being of parents, particularly with mothers and in relation to maternal depression, and on specific coping styles [29]. However, the challenge remains for researchers to isolate the respective contribution of the families’ impoverished experiences to their state of well-being when these poverty-related forces impinging on their lives are dynamic and interactive in nature. Socioeconomic disadvantage in childhood is evidently related to both immediate and persisting impairments in mental health and well-being [23,30]. Family processes and family stress models have shown children and adolescents who grow up in families in poverty to have both more internalizing and externalizing symptoms compared with their peers raised in more affluent families [31]. The level and stability of family income have distinct effects on family functioning and children’s well-being [32,33].

### Aims of This Study

The study aims to examine the following research questions:

1. To elucidate the *Family Agency* construct through interviews with mother-child dyads (qualitative data).
2. How are financial factors, including financial stress and resources, differentially related to home environment, parent stress, and parent and child outcomes?
3. Does *Family Agency* mediate the relationship between financial stress, internal and external resources, and home environment, which result in better parents’ and children’s outcomes?
4. Are there subgroups of children with distinct trajectories in terms of the levels of positive *Family Agency* perceived by the participants? How do they differ in terms of home environment and parent stress factors? Are there significantly different outcomes experienced by the children in each subgroup over 3 time points? Do the family outcomes differ significantly?
5. What is the strength of the relationships among financial stress, internal and external resources, home environment, parental stress, and outcomes according to gender and ethnicity?
6. What are the characteristics of the microagentic processes and the dynamics of intersections between families and the external ecological environment among families with positive children, parents, and family outcomes based on 2 SD above mean versus with outcomes 2 SD below mean scores?

## Methods

### Overview

This study will employ a sequential longitudinal mixed-methods design, which consists of 2 phases of data collection. In phase 1, in-depth interviews will be conducted with 60 mother-child dyads. To examine the adaptive processes of different families facing financial stress, maximum variant sampling criteria will be utilized to include (1) ethnicity (the 3 major ethnic groups of Chinese, Malays, and Indian families); (2) genders of children; and (3) family types: intact, single parent, and stepfamily. The mothers and children will be asked to describe their relationship with one another and share with the interviewers’ things that worry them the most and how they formulate solutions to these challenges. On the basis of the results from the 120 interviews, the team will operationalize and develop measures for the construct of *Family Agency* guided by the SRT.

In phase 2 of the study, we collect repeated measures longitudinal survey data over 3 time points. A total of 800 pairs of mother-child dyads with children aged between 7 and 12 years whose families are receiving ComCare financial assistance will be invited to participate in this longitudinal study. Phase 2 tests the conceptual framework of this proposed study, built on known risk factors of children growing up in families facing financial stress, together with the measurements of *Family Agency* developed in phase 1. On the basis of the findings of the longitudinal data, 10 focus groups discussions with up to 15 participants in each group will be held after each preliminary data analysis is completed to validate the findings. These 150 participants will be drawn from the participants of phase 2a of this study.

**Figure 1:**
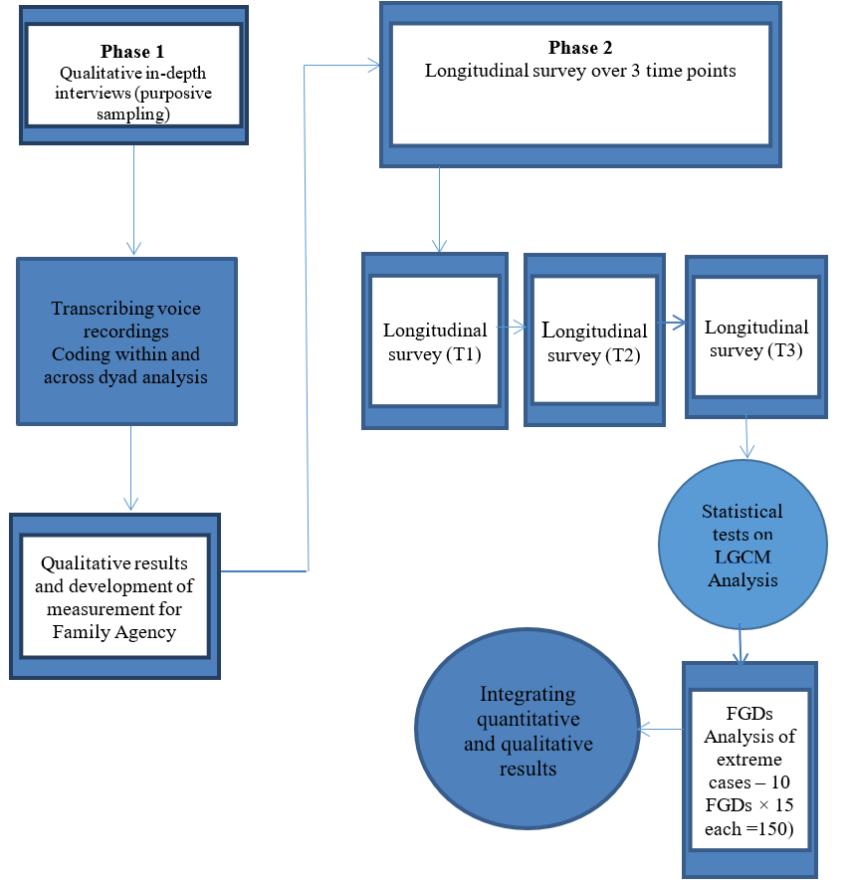
Schematic design.

Figure 1 gives an overview of the data collection methods that will be used in this study.

### Ethics

This study has been approved by the Institutional Review Board at the National University of Singapore (S-18-003).

### Participants

Singaporean families with 1 or more children aged between 7 and 12 years and currently receiving government financial assistance are eligible to participate in this study. The mother and 1 child within the mentioned age range will be recruited from each participating family. To examine the adaptive processes of different families facing financial stress, maximum variant sampling criteria will be utilized to include (1) 4 major ethnic groups: Chinese, Malay, Indian, and others; (2) both genders of children aged between 7 and 12 years; and (3) different family types: intact, single parent, and stepfamily.

### Recruitment Process

#### Phase 1

The officers from the Ministry of Social and Family Development will identify recipients of ComCare financial assistance that fit into the recruitment criteria. A letter of invitation to participate in this study has been sent out to all these families. To expedite the recruitment process, 2 part-time research assistants (RAs) have been hired to telephone potential participants from the sampling list to attain the desired sample size. In addition to phone calls, the RAs will make home visits to potential participants by knocking on doors during the recruitment.

Verbal consent will first be obtained when the families indicate interest to participate. Written consent will be obtained from both mother and child when the researchers visit the families. The RAs will also spend time to break the ice and build trust with the participants. The child and mother dyad will be interviewed separately. Children and mothers will be assured that the information provided by one party will not be disclosed to the other to maintain confidentiality.

#### Phase 2a

Approximately 800 mother-child dyads from the same source will be sampled, based on the expected correlation of the variable (*ρ*), type 1 error, power, extent of measurement errors (σ^2^), within-subject variance (s_x_^2^), and the smallest meaningful differences are set at 0.6, 0.05, 0.8, 0.8, 0.6, and 0.05, respectively. This sample size is based on longitudinal sample size consideration [34]. Survey interviews will be conducted with the same sample at 3 different points in time with a 6-month interval between waves of data collections. The feedback from review panel has been provided as Multimedia Appendix 1. The researchers will visit the mothers and children of the families to obtain consent. They will be informed that it is a 3-wave data collection with intervals of 6 months, and the same interviewer will be conducting the 3 waves of survey with the same families to facilitate building trust and rapport. To minimize attrition rates, tokens of appreciation will be presented to the mother and children after each wave of survey completion. The RAs will also send festive greeting cards to keep in touch with the participants. The mothers and children will also be informed that at the end of the 3 waves of survey, they may be invited to focus group discussions (FGDs) to provide inputs to the analysis of the findings. They are considered the experts of their own lives and are in the best position to validate the findings.

#### Phase 2b

After the initial data analysis of phase 2a, the major trajectories of adaptive behaviors will be identified among the samples. According to the trajectories identified, focus groups will be formed. From the original sampled families, the research team will invite participants from similar trajectories (outcome measures) for the specific focus groups to collect their input for the analysis. It is estimated that up to 150 participants will take part in the focus groups.

### Measures

For covariates including gender, ethnicity, citizenship, and educational levels of mothers and children, a demographic form will be filled in to capture the basic information. The *antecedents* include the financial stress and resources of the family, which will be measured by the *Economic Hardship Questionnaire* [35] consisting of 12 items focusing on financial conditions experienced by the family in the past 6 months and family resilience, which will be measured by the 10-item *Family Hardiness Scale* [36]. The construct refers to the internal strengths and durability of the family unit and is characterized by a sense of control over the outcomes of life events and hardships, a view of change as beneficial and growth producing, and an active rather than passive orientation in adjusting to and managing stressful situations. Intermediary constructs include *Family Agency*, which will be measured by the scale we would develop in phase 1; *Parents’ Efficacy* measured by the *Adult Hope Scale*-12 items [37]; *Children’s Efficacy* measured by the *Children Hope Scale* consisting of 6 items tapping agency and pathways of agency [38]; and *Home Environment* construct measures how families tap on resources within the home, which serves as sources of strength and support to its family members. This is measured by the *Family Environment Scale* [39], which has 45 items measuring 2 dimensions of family relationships and systems maintenance. *Outcome variables* include *Children’s outcomes*, which is measured by the *Behavior Assessment System for Children* [40] and self-assessment of health condition using EQ-5D-Y [41]. *Parents’ Outcomes* will be measured by the DASS [42], which examines parent health by assessing the extent to which parents experience life stresses, particularly under economic stress, and *Brief COPE Scale* [43], consisting of 28 items assessing specific adaptive and maladaptive coping strategies and EQ-5D [41].

These scales are selected as they have been used by researchers examining low-income families. This will facilitate the comparison of results. We will conduct separate Confirmatory Factor Analyses to validate the *a priori* factor structures of all measures. All the scales will be translated into Mandarin, Malay, and Tamil as we expect a good proportion of the families from different ethnic groups to be more conversant in these languages. The constructs will be measured with self-reporting questionnaires to be completed by parent and child. The questionnaires used in this study have been provided as Multimedia Appendix 2.

### Data Analysis

#### Data Analysis Plans for Phase 1

The data analysis at this phase aims to stay close to the data, which are a low-level interpretation with the goal to understand the latent variables closely related to the construct of Family Agency. Thematic coding will be performed on all the transcripts of the semistructured interviews with the aid of qualitative software QSR Nvivo 11. Quasi-statistical analysis methods will be used to summarize data with descriptive statistics for concept clarification on the construct *Family Agency* and instrument development.

#### Data Analysis Plans for Phase 2a

Sequential Equation Model (SEM) will be used to analyze the data collected at the 3 time points. The estimated coefficients of SEM will provide the magnitudes of the level of influences on the various constructs on child and family outcomes. Descriptive statistics such as Pearson correlational analyses will be carried out to examine the degree of association of financial stress, internal and external resources, home environment, parental stress, and the 2 outcomes, breakdown by gender and ethnicity by chi-square. As gender and ethnicity are discrete variables and the number of groups is relatively small, multiple-group longitudinal SEM will be carried out to test whether the regression coefficients differ for gender and ethnicity. Factorial invariance across groups will be examined before proceeding with multiple-group longitudinal SEM. The existence of different trajectories in these low-income families will be examined using the Latent Growth Curve Model (LGCM) to find out whether heterogeneity exists for the families. Factor scores will be generated for the dimensions of the *Family Agency* construct over the 3 waves. The mediating role of *Family Agency* will be investigated in 2 ways. First, path analysis approach using SEM will be used to examine the fit between *Family Agency* as an intermediate between financial stress “and” or “or” resources. Model fit will be examined by the Root Means Squared Error of Approximation, Comparative Fit Index, Akaike’s information criterion, and Bayes information criterion. Evidence of a mediating role of *Family Agency* will be defined by statistically significant (*P*<.05) coefficients for paths between independent variables (financial stress and resources) and *Family Agency* and between *Family Agency* and the dependent variables (child outcome and parent outcome). Second, if statistically significant paths are identified, the potential causal role of *Family Agency* will be investigated via a doubly robust propensity score approach, inverse probability-weighted regression adjustment [44,45]. Briefly, the approach proceeds as follows: the proportion of the total effects of the independent variables because of controlled direct effects and natural indirect effects (as mediated through Family Agency) will be estimated using a system of 2 multivariable logistic regressions (1 for the mediator and 1 for the outcome) weighted by the joint inverse probability of the independent variable and mediator (Family Agency). These equations will be used to predict outcomes under different levels of treatment, with the difference between predicted outcomes providing estimates of the direct and indirect effects using regression adjustment. These methods are shown to be doubly robust to misspecification of either the exposure or outcome models and thus less vulnerable to residual confounding [46]. As *Family Agency* may also modify the relationship between independent variables on outcomes, we will also investigate the presence of interaction in estimated mediation models [44].

#### Phase 2b: Focus Group Discussions

On the basis of these identified trajectories, qualitative data through FGDs (n=10) will be used to conduct contrasting case analysis. Moreover, 2 FGDs for each trajectory will be conducted separately with children and mothers (2 FGDs × 5 trajectories=10 FGDs with up to 15 members each) and will be used to closely examine the contrasting family processes between highest and lowest score cases. Group comparisons across ethnicities, family types, and family size among the positive deviance sample will be examined.

### Sample Size and Power Calculations

#### Sample Size Justification for Longitudinal Survey Data

A total of 800 children aged between 7 and 12 years and their mothers matching the sampling criteria stated in phase 1 will be invited to participate. The sample size of 800 is based on the degree of accuracy of 0.034 [47]. Survey interviews will be conducted with the same sample at 3 different points in time with 6-month intervals between waves of data collections. This sample size of 800 is based on longitudinal sample size consideration [43]. The expected correlation of the variable (*ρ*), type 1 error, power, extent of measurement errors (σ^2^), within-subject variance (s_x_^2^), and smallest meaningful difference (d) are set at 0.6, 0.05, 0.8, 0.8, 0.6, and 0.05 respectively. Package R longpower, liu.liang.linear.power function, is used to calculate the sample.

#### Phase 1: Sample Size n=60 (Mother-Child Dyads)

This phase aims to obtain an intimate understanding of the microprocesses of low-income families. A total of 60 mothers and children will be interviewed separately with a semistructured guide. Children should be aged between 7 and 12 years and their families should be current recipients of financial aid from ComCare.

#### Phase 2a: Sample Size n=800 for 3 Waves of Surveys

Children (n=800) together with their mothers (n=800) who fulfill the sampling criteria stated in phase 1 will be invited to participate. Survey interviews will be conducted with the same sample at 3 different points, with a 6-month interval before the next wave of data collection. The sample size determination has been provided in Table 1.

**Figure 2:**
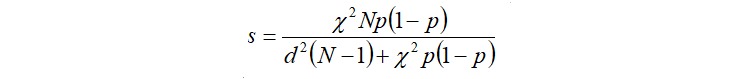
Calculation of sample size.

Calculation for sample size has been illustrated in Figure 2, where *s* is the required sample size; *X^2^* is chi square for *df*=1 (3.841); *N* is population size; *p* is population proportion (assumed 0.5); and *d* is degree of accuracy expressed as a proporation.

### Strategies to Minimize Attrition Rates

One of the biggest challenges in collecting longitudinal data is the loss of contact over several time points, which will compromise the validity and integrity of the study [48]. A 2-pronged protocol will be employed in this proposed study to minimize dropout.

#### Prong 1: Researcher-Oriented Strategies

The 800 child-mother dyads will be interviewed by 40 trained interviewers who will follow the same families over the 3 waves of interviews. Each interviewer will follow up with 20 child-mother dyads. All interviewers have to undergo intensive training in engaging low-income families, rapport building, interview skills, interview ethics, role playing, handling confidentiality, and cultural sensitivity, conducted over 3 weekends (15 hours in total). Systematic training manual documenting the protocols will be utilized to guide the interviewers.

#### Prong 2: Participant-Oriented Strategies

In the 6-month gap between the waves of the interviews, the interviewers will make 1 call to each family per month and send festive greeting cards to keep in touch. The token of appreciation per dyad is incremental across progress time points to incentivize the participants to stay in the study. Nevertheless, the overall dropout rate is estimated to be 10 ± 5% [49]. The statistical difference between lost-to-follow-up dyad and those remaining in the study will be assessed using appropriate methods (eg, chi-square), regarding input/outcome variables pertinent to the objectives/hypothesis of this study.

**Table 1:** Sample size determination based on degree of accuracy and population size.

Particular	Population (n=10,000)	Population (n=20,000)
Degree of accuracy	0.033	0.034
Sample size	810	798

## Results

Phase 1 recruitment has achieved the planned 60 dyads as on October 1, 2018. Interviews will start in November 2018 and continue until February 2019. The development of the measurement scale for the *Family Agency* construct will be developed and piloted between March and May 2019. Moreover, 3 waves of survey will be implemented between May 2019 and July 2020. Data analysis will be performed for every wave. The statistical analysis to identify the trajectories of adaption will take place concurrently. FGDs will be held in August 2020. The final analysis and integration of results will be carried out by the research team between September 2020 and January 2021. These results will be written for publications between February and May of 2021 before the project concludes.

## Discussion

### Principal Findings

This proposed study aims to contribute to family research scholarship on 2 fronts: *conceptual advancement* and *longitudinal empirical evidence*. These advancements in concept and datasets will inform and facilitate the *shift in policy and practice* with low-income families. In this proposed study, the team plans to conduct a short-term 3-wave longitudinal survey over 18 months. The major benefit of using a 3-wave longitudinal study for this project is the advantage of detecting developments and changes of *Family Agency* and adaptive capacities over time. Conducting 3 waves is the minimum period that allows the application of LGCM to examine whether these lower-income families grow, decline, or remain stable in their Family Agency. LGCM focuses on within-individual changes, resulting in more accurate and nuanced conclusions concerning the adult and child outcomes [50]. More importantly, predictor variables and their consequences are built into the model [51]. Thus, it allows us to investigate different growth parameters and the incorporation of both time-varying predictors (eg, child and parent hope) and time-invariant predictors (eg, gender and race). Establishing the best possible causal effect is made possible through LGCM, whereas a cross-sectional design does not allow this. For instance, how the effect of financial stress of parents at first wave might affect parenting in the second wave. Without 3 waves of data collection design, the taxonomy on the *Family Agent* would be a static concept. While examining the changes over the 3 waves using a latent-class growth model, the dynamics of *Family Agency* are incorporated into the taxonomy.

### Contributions to Policy and Practice

Existing local, economic, and social policy researchers [52-55] have underscored the risk of a rich-poor divided Singapore and the pileup risks confronted by poor families [2,56] and have advocated for decisive corrective measures. Results of these studies by economists are valuable in providing a clearer problem definition to policy makers, which may have contributed, at least in part, to the move from the traditionally welfare-aversive government stance to one that actively puts in place measures to promote inclusive growth and strengthen its redistributive role [57].

This meticulous examination of social problems to achieve a clear problem definition as the base of policy construction has to be balanced with similar attention in assessing the strengths of the low-income families and the environment that the policies target. Indeed, emerging behavioral economics research has shown that resource availability may be insufficient to *move* families out of poverty and that attention should instead be given to default adaptive processes that some families use to help them rise above their economic and social situations [58,59]. In other words, locating change factors at the individual family level helps to identify the impetus for sustainable practices within lived contexts and helps us to understand what low-income families draw on to foster their hope and optimism amidst multiple impositions and constraints.

## Appendix

Multimedia Appendix 1 Peer-reviewer report from Social Science Research Council Singapore.

Multimedia Appendix 2 Questionnaires used in this study.

## Abbreviations

DASS: Depression, Anxiety and Stress Scale
FGD: focus group discussion
LGCM: Latent Growth Curve Model
RA: research assistant
SEM: Sequential Equation Model
SRT: Social Relational Theory
