# Supplementary material for: Identifying Positive Adaptive Pathways in Low-Income Families in Singapore: Protocol for Sequential, Longitudinal Mixed-Methods Design
Source: JMIR Res Protoc. 2019 Feb 1;8(2):e11629. doi: 10.2196/11629 (PMC6376333; doi:10.2196/11629)
Supplement: Multimedia Appendix 1 [file resprot_v8i2e11629_app1.pdf]

<u>MOE2016-SSRTG-039</u>
Project title: Identifying Positive Adaptive Pathways in Low-income families in Singapore
PI: A/Prof Esther Goh Chor Leng

Review Comments:
The project has the potential for real impact in improving the welfare of low-income families in Singapore.

Regarding the proposal for a three-wave, 18 month longitudinal study for phase 2, the panel suggested that the Principal Investigator could consider stretching the duration so that there would be sufficient variation in the data for analysis.

It was also highlighted that some social policies might have an effect on the adaption process in the target group, and these should be addressed as potential variables for the study.
