# Supplementary material for: Identifying Positive Adaptive Pathways in Low-Income Families in Singapore: Protocol for Sequential, Longitudinal Mixed-Methods Design
Source: JMIR Res Protoc. 2019 Feb 1;8(2):e11629. doi: 10.2196/11629 (PMC6376333; doi:10.2196/11629)
Supplement: Multimedia Appendix 2 [file resprot_v8i2e11629_app2.pdf]

Study ID:
Date:

<u>Questionnaires used in the study</u>

1. Economic Hardship Questionnaire

2. Family Hardiness Scale

3. Adult Hope Scale

4. Children Hope Scale

6. Family Environment scale

5. BASC

6. EQ5DY

7. Brief COPE

8. DASS

Study ID:
Date:

Economic Hardship Questionnaire

For the following 10 questions choose one of the answers listed below: A = never C = often B = sometimes D = very often

During the last 6 months, how often did your family:

1. Cut back on social activities and entertainment expenses? A B C D

2. Postpone major household purchases? A B C D

3. Postpone clothing purchases? A B C D

4. Change transportation patterns to save money? A B C D

5. Change food shopping or eating habits to save money? A BC D

6. Cut back on charitable contributions? A B C D

7. Reduce household utility use? A B C D

8. Sell some possessions? A B C D

9. Postpone medical care to save money? A B C D

10. Take additional employment to help meet expenses? A B C D

11. Which of the following best described what has happened to your family income during the past 6 months?

A. Has increased very much; B. Has increased somewhat; C. Has stayed the same; D. Has decreased somewhat; E. Has decreased very much.

12. Which of the following best describes your family financially at this time:

A. No problems B. Minor problems C. Major problems D. Extreme problems

Study ID:
Date:

Family Hardiness Scale for Mothers

Instructions: Please read each statement below and decide to what extent each best describes your family. Is the statement False (0), Mostly False (1), Mostly True (2) or Totally True (3)? Circle the number that best matches your feelings about each statement. Please respond to each and every statement.

In Our Family…	Does not apply	False	Mostly False	Mostly True	True
1. In the long run, the bad things that happen to us are balanced by the good things that happen.					
2. We have a sense of being strong even when we face big problems.					
3. While we don't always agree, we can count on each other to stand by us in times of need.					
4. We believe that things will work out better if we work together as a family.					
5. We strive together and help each other no matter what.					
6. When our family plans activities we try new and exciting things.					
7. We listen to each others' problems, hurts and fears.					
8. We seem to encourage each other to try new things and experiences.					
9. Being active and learning new things are encouraged.					
10. We work together to solve problems.					

Copyright 1986. Marilyn A. McCubbin and Hamilton I McCubbin. All rights reserved.

Family Quality of Life Index

People often times feel different about their family's quality of life. Please indicate which rating best describes how well your family's life is going at this time.

Worst Life Possible				Surviving life					Best Life Possible
1	2	3	4	5	6	7	8	9	10

Study ID:
Date:

The Trait Hope Scale for Mothers (Adult Hope Scale)

Directions: Read each item carefully. Using the scale shown below, please select the number that best describes YOU and put that number in the blank provided.

1. = Definitely False (100% Not True)
2. = Mostly False (75% Not True)
3. = Somewhat False (50% Not True)
4. = Slightly False (25% Not True)
5. = Slightly True (25% True)
6. = Somewhat True (50% True)
7. = Mostly True (80% True)
8. = Definitely True (100% True)

____ 1. I can think of many ways to get out of a traffic jam.

____ 2. I energetically pursue my goals.

____ 3. I feel tired most of the time.

____ 4. There are lots of ways around any problem.

____ 5. I am easily downed in an argument.

____ 6. I can think of many ways to get the things in life that are important to me.

____ 7. I worry about my health.

____ 8. Even when others get discouraged, I know I can find a way to solve the problem.

____ 9. My past experiences have prepared me well for my future.

____10. I've been pretty successful in life.

____11. I usually find myself worrying about something.

____12. I meet the goals that I set for myself.

Study ID:
Date:

Questions About Your Goals (for children) (Children Hope Scale)

Instructions: The six sentences below describe how children think about themselves and how they do things in general. Read each sentence carefully. For each sentence, please think about how you are in most situations. Place a check inside the circle that describes YOU the best. For example, place a check (√) in the circle (O) above "None of the time," if this describes you. Or, if you are this way "All of the time," check this circle. Please answer every question by putting a check in one of the circles. There are no right or wrong answers.

1. I think I am doing pretty well.

O	O	O	O	O	O
None of the time	A little of the time	Some of the time	A lot of the time	Most of the time	All of the time

2. I can think of many ways to get the things in life that are most important to me.

O	O	O	O	O	O
None of the time	A little of the time	Some of the time	A lot of the time	Most of the time	All of the time

3. I am doing just as well as other kids my age.

O	O	O	O	O	O
None of the time	A little of the time	Some of the time	A lot of the time	Most of the time	All of the time

4. When I have a problem, I can come up with lots of ways to solve it.

O	O	O	O	O	O
None of the time	A little of the time	Some of the time	A lot of the time	Most of the time	All of the time

5. I think the things I have done in the past will help me in the future.

O	O	O	O	O	O
None of the time	A little of the time	Some of the time	A lot of the time	Most of the time	All of the time

6. Even when others want to quit, I know that I can find ways to solve the problem.

O	O	O	O	O	O
None of the time	A little of the time	Some of the time	A lot of the time	Most of the time	All of the time

The Family Environment Scale (published by Mind Garden, http://www.mindgarden.com) is composed of 10 subscales that measure the actual, preferred, and expected social environments of all types of families. This scale is a paid scale and the sample is unavailable online. The 10 subscales assess three sets of dimensions: (a) relationship dimensions (cohesion, expressiveness, conflict); (b) personal growth or goal orientation dimensions (independence, achievement orientation, intellectual-cultural orientation, active-recreational orientation, moral-religious emphasis); and (c) system maintenance dimensions (organization, control). The relationship and system maintenance dimensions primarily reflect internal family functioning; the personal growth or goal orientation dimensions primarily reflect the linkages between the family and the larger social context.

The FES has three forms:

1.The Real Form (Form R) measures people's perceptions of their current family or their family of origin. This form is used to assess individuals' perceptions of their conjugal and nuclear families, formulate clinical case descriptions, monitor and promote improvement in families, focus on how families adapt to life transitions and crises, understand the impact of the family on children and adolescents, and predict and measure the outcome of treatment.

2.The Ideal Form (Form I) measures people's preferences about an ideal family environment. This form is used to measure family members' preferences about how a family should function; assess family members' value orientations and how they change over time, such as before and after family counseling; and identify areas in which people want to change their family.

3.The Expectations Form (Form E) measures people's expectations about family settings. This form is used in premarital counseling to clarify prospective partners' expectations of their family, help members of blended families describe how they expect their new family to function, and identify parents' expectations about their family after a major life transition, such as retirement or the youngest child's leaving home.

The FES manual presents normative data on 1,432 normal families and 788 distressed families, describes the derivation and application of a family incongruence score that assesses the extent of disagreement among family members, presents psychometric information on the reliability and stability of the subscales, and covers the research applications and validity of the subscales. The manual includes a conceptual model of the determinants and outcomes of the family environment and reviews studies focusing on families of youth with behavioral, emotional, or developmental disabilities; families with a physically ill child; families with a history of physical or sexual abuse; and families of patients with medical and psychiatric disorders. The FES has also been used to focus on the relationship between the family environment and child development and adult adaptation and on families coping with life transitions and crises, such as parent or child death, unemployment and economic deprivation, immigration and acculturation, and combat and war.

Study ID:
Date:

Behaviour Assessment System for Children- BASC 3

Section 1

Instructions: This first section contains sentences that tell how some boys and some girls think or feel or act. Read each sentence carefully. For this section, you will have two answer choices: **T or F.**

Circle **T** for **True** if you agree with a sentence.

Circle **F** for **False** if you do not agree with a sentence.

1. I like who I am **T F**	11. My parents blame too many of their problems on me **T F**
2. My friends have more fun than I do **T F**	12. Things go wrong for me, even when I try **T F**
3. I have never been in a car **T F**	13. Other children don't like to be with me **T F**
4. I don't care about school. **T F**	14. I think I have short attention span **T F**
5. I never get my way **T F**	15. My classmates don't like me **T F**
6. I have a hard time making friends **T F**	16. I can't seem to control what happens to me **T F**
7. I feel good about myself. **T F**	17. I can't wait for school to be over. **T F**
8. I don't like thinking about school. **T F**	18. I wish I were different **T F**
9. I never seem to get anything right **T F**	19. I often do things without thinking **T F**
10. I have attention problems **T F**	

Section 2

For the second section, you will have four answer choices to choose from: **N, S, O, and A.**

Circle **N** if the sentence **never** describes you or how you feel.

Circle **S** if the sentence **sometimes** describes you or how you feel.

Circle **O** if the sentence **often** describes you or how you feel.

Circle **A** if the sentence **almost always** describes you or how you feel.

20. My school feels good to me.	**N S O A**
21. I am left out of things	**N S O A**
22. When I take tests, I can't think.	**N S O A**
23. I am lonely.	**N S O A**
24. I forget to do things.	**N S O A**
25. I'm happy with who I am	**N S O A**
26. People get mad at me, even when I don't do anything wrong	**N S O A**

27. People tell me that I am too noisy	**N**	**S**	**O**	**A**
28. People tell me to try harder	**N**	**S**	**O**	**A**
29. I get into trouble for not paying attention	**N**	**S**	**O**	**A**
30. My mother and father help me if I ask them to	**N**	**S**	**O**	**A**
31. My looks bother me.	**N**	**S**	**O**	**A**
32. I hate school.	**N**	**S**	**O**	**A**
33. I talk without waiting for others to say something	**N**	**S**	**O**	**A**
34. I feel safe at school.	**N**	**S**	**O**	**A**
35. I listen when people are talking to me	**N**	**S**	**O**	**A**
36. I am proud of my parents.	**N**	**S**	**O**	**A**
37. I have trouble sitting still	**N**	**S**	**O**	**A**
38. I like the way I look	**N**	**S**	**O**	**A**
39. I get blamed for things I can't help	**N**	**S**	**O**	**A**
40. My parents like to be with me	**N**	**S**	**O**	**A**
41. I am liked by others.	**N**	**S**	**O**	**A**
42. I want to do better but I can't	**N**	**S**	**O**	**A**
43. I like my parents	**N**	**S**	**O**	**A**
44. My mother and father like my friends.	**N**	**S**	**O**	**A**
45. Other people seem to ignore me	**N**	**S**	**O**	**A**
46. School is boring.	**N**	**S**	**O**	**A**
47. Even when I try hard, I fail	**N**	**S**	**O**	**A**
48. My parents are proud of me	**N**	**S**	**O**	**A**
49. People say bad things to me	**N**	**S**	**O**	**A**
50. I am blamed for things I don't do.	**N**	**S**	**O**	**A**
51. I have trouble paying attention to the teacher	**N**	**S**	**O**	**A**
52. It is hard for me to keep my mind on school work	**N**	**S**	**O**	**A**
53. I am disappointed with my grades	**N**	**S**	**O**	**A**
54. I am a good listener	**N**	**S**	**O**	**A**
55. I get along well with others	**N**	**S**	**O**	**A**
56. I am bothered by teasing from others	**N**	**S**	**O**	**A**

57. My parents trust me	**N S O A**
58. I feel nobody likes me.	**N S O A**
59. Other people find things wrong with me	**N S O A**
60. My parents listen to what I say.	**N S O A**
61. Other people make fun of me.	**N S O A**
62. I talk while other people are talking	**N S O A**
63. I feel out of place around people	**N S O A**
64. I have trouble standing still in lines	**N S O A**
65. I get upset about my looks	**N S O A**
66. I feel like I want to quit school.	**N S O A**
67. I have trouble paying attention to what I am doing	**N S O A**
68. My parents are easy to talk to	**N S O A**
69. I fail at things	**N S O A**
70. People tell me to slow down	**N S O A**
71. People act as if they don't hear me	**N S O A**
72. Other kids hate to be with me.	**N S O A**
73. My parents expect too much from me	**N S O A**
74. People tell me to be still	**N S O A**
75. I like going places with my parents	**N S O A**

Study ID:
Date:

Sample

Health Questionnaire

English version for the UK

UK (English) © 2008 EuroQol Group EQ-5D™ is a trade mark of the EuroQol Group

Study ID:
Date:

EQ-5D-Y

Describing your health TODAY

Under each heading, please tick the ONE box that best describes your health TODAY.

Mobility *(walking about)*

I have no problems walking about ☐
I have some problems walking about ☐
I have a lot of problems walking about ☐

Looking after myself

I have no problems washing or dressing myself ☐
I have some problems washing or dressing myself ☐
I have a lot of problems washing or dressing myself ☐

Doing usual activities *(for example, going to school, hobbies, sports, playing, doing things with family or friends)*

I have no problems doing my usual activities ☐
I have some problems doing my usual activities ☐
I have a lot of problems doing my usual activities ☐

Having pain or discomfort

I have no pain or discomfort ☐
I have some pain or discomfort ☐
I have a lot of pain or discomfort ☐

Feeling worried, sad or unhappy

I am not worried, sad or unhappy ☐
I am a bit worried, sad or unhappy ☐
I am very worried, sad or unhappy ☐

Sample

Study ID:
Date:

How good is your health TODAY

- We would like to know how good or bad your health is TODAY.
- This line is numbered from 0 to 100.
- 100 means the best health you can imagine.
  0 means the worst health you can imagine.
- Please mark an X on the line that shows how good or bad your health is TODAY.

The best health
you can imagine

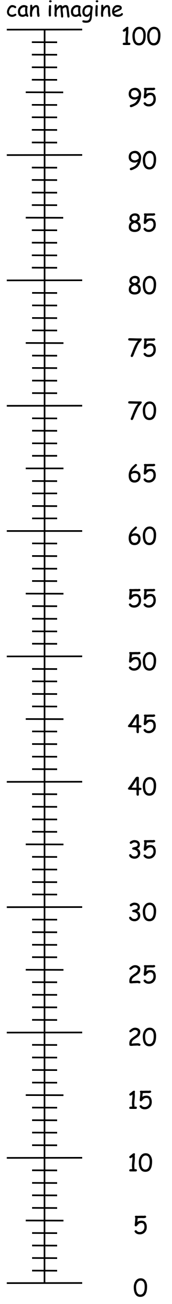

The worst health
you can imagine

Sample

Study ID:
Date:

Brief COPE

These items deal with ways you've been coping with the stress in your life since you found out you were going to have to have this operation. There are many ways to try to deal with problems. These items ask what you've been doing to cope with this one. Obviously, different people deal with things in different ways, but I'm interested in how you've tried to deal with it. Each item says something about a particular way of coping. I want to know to what extent you've been doing what the item says. How much or how frequently. Don't answer on the basis of whether it seems to be working or not—just whether or not you're doing it. Use these response choices. Try to rate each item separately in your mind from the others. Make your answers as true FOR YOU as you can.

1 = I haven't been doing this at all
2 = I've been doing this a little bit
3 = I've been doing this a medium amount
4 = I've been doing this a lot

1. I've been turning to work or other activities to take my mind off things.
2. I've been concentrating my efforts on doing something about the situation I'm in.
3. I've been saying to myself "this isn't real.".
4. I've been using alcohol or other drugs to make myself feel better.
5. I've been getting emotional support from others.
6. I've been giving up trying to deal with it.
7. I've been taking action to try to make the situation better.
8. I've been refusing to believe that it has happened.
9. I've been saying things to let my unpleasant feelings escape.
10. I’ve been getting help and advice from other people.
11. I've been using alcohol or other drugs to help me get through it.
12. I've been trying to see it in a different light, to make it seem more positive.
13. I’ve been criticizing myself.
14. I've been trying to come up with a strategy about what to do.
15. I've been getting comfort and understanding from someone.
16. I've been giving up the attempt to cope.
17. I've been looking for something good in what is happening.
18. I've been making jokes about it.
19. I've been doing something to think about it less, such as going to movies, watching TV, reading, daydreaming, sleeping, or shopping.
20. I've been accepting the reality of the fact that it has happened.
21. I've been expressing my negative feelings.
22. I've been trying to find comfort in my religion or spiritual beliefs.
23. I’ve been trying to get advice or help from other people about what to do.
24. I've been learning to live with it.
25. I've been thinking hard about what steps to take.
26. I’ve been blaming myself for things that happened.
27. I've been praying or meditating.
28. I've been making fun of the situation.

Study ID:
Date:

DASS21

Name: *Date:*

Please read each statement and circle a number 0, 1, 2 or 3 which indicates how much the statement applied to you *over the past week*. There are no right or wrong answers. Do not spend too much time on any statement.

The rating scale is as follows:

0 Did not apply to me at all
1 Applied to me to some degree, or some of the time
2 Applied to me to a considerable degree, or a good part of time
3 Applied to me very much, or most of the time

1	I found it hard to wind down	0	1	2	3
2	I was aware of dryness of my mouth	0	1	2	3
3	I couldn't seem to experience any positive feeling at all	0	1	2	3
4	I experienced breathing difficulty (eg, excessively rapid breathing, breathlessness in the absence of physical exertion)	0	1	2	3
5	I found it difficult to work up the initiative to do things	0	1	2	3
6	I tended to over-react to situations	0	1	2	3
7	I experienced trembling (eg, in the hands)	0	1	2	3
8	I felt that I was using a lot of nervous energy	0	1	2	3
9	I was worried about situations in which I might panic and make a fool of myself	0	1	2	3
10	I felt that I had nothing to look forward to	0	1	2	3
11	I found myself getting agitated	0	1	2	3
12	I found it difficult to relax	0	1	2	3
13	I felt down-hearted and blue	0	1	2	3
14	I was intolerant of anything that kept me from getting on with what I was doing	0	1	2	3
15	I felt I was close to panic	0	1	2	3
16	I was unable to become enthusiastic about anything	0	1	2	3
17	I felt I wasn't worth much as a person	0	1	2	3
18	I felt that I was rather touchy	0	1	2	3
19	I was aware of the action of my heart in the absence of physical exertion (eg, sense of heart rate increase, heart missing a beat)	0	1	2	3
20	I felt scared without any good reason	0	1	2	3
21	I felt that life was meaningless	0	1	2	3
